# Prognostic accuracy of the one-legged balance test in predicting falls: 15-years of midlife follow-up in a British birth cohort study

**DOI:** 10.3389/fspor.2022.1066913

**Published:** 2023-01-09

**Authors:** Joanna M. Blodgett, Rebecca Hardy, Daniel H. J. Davis, Geeske Peeters, Mark Hamer, Diana Kuh, Rachel Cooper

**Affiliations:** ^1^ Institute of Sport, Exercise & Health, Division of Surgery & Interventional Science, University College London, London, UK; ^2^ School of Sport, Exercise and Health Sciences, Loughborough University, Loughborough, UK; ^3^ Social Research Institute, University College London, London, UK; ^4^ MRC Unit for Lifelong Health and Ageing, UCL, London, UK; ^5^ Department of Geriatric Medicine, Radboud University Medical Centre, Nijmegen, Netherlands; ^6^ Department of Sport and Exercise Sciences, Musculoskeletal Science and Sports Medicine Research Centre, Manchester Metropolitan University Institute of Sport, Manchester, UK; ^7^ AGE Research Group, Translational and Clinical Research Institute, Faculty of Medical Sciences, Newcastle University, Newcastle Upon Tyne, UK; ^8^ NIHR Newcastle Biomedical Research Centre, Newcastle University and Newcastle upon Tyne Hospitals NHS Foundation Trust, Newcastle Upon Tyne, UK

**Keywords:** one-legged balance, falls, Fall history, life course, risk prediction

## Abstract

**Introduction:**

The one-legged balance test is a common screening tool for fall risk. Yet, there is little empirical evidence assessing its prognostic ability. The study aims were to assess the prognostic accuracy of one-legged balance performance in predicting falls and identify optimal cut-points to classify those at greater risk.

**Methods:**

Data from up to 2,000 participants from a British birth cohort born in 1,946 were used. The times an individual could stand on one leg with their eyes open and closed were recorded (max: 30 s) at ages 53 and 60–64. Number of falls in the past year was self-reported at ages 53, 60–64 and 68; recurrent falls (0–1 vs. 2+) and any fall (0 vs. 1+) were considered binary outcomes. Four longitudinal associations between balance times and subsequent falls were investigated (age 53 → 60–64; age 53 → 68; age 60–64 → 68; age 53 & 60–64 → 68). For each temporal association, areas under the curve (AUC) were calculated and compared for a base sex-only model, a sex and balance model, a sex and fall history model and a combined model of sex, balance and fall history. The Liu method was used to identify optimal cut-points and sensitivity, specificity, and AUC at corresponding cut-points.

**Results:**

Median eyes open balance time was 30 s at ages 53 and 60–64; median eyes closed balance times were 5 s and 3 s, respectively. The predictive ability of balance tests in predicting either fall outcome was poor (AUC range for sex and balance models: 0.577–0.600). Prognostic accuracy consistently improved by adding fall history to the model (range: 0.604–0.634). Optimal cut-points ranged from 27 s to 29 s for eyes open and 3 s to 5 s for eyes closed; AUC consistently indicated that using “optimal” cut-points to dichotomise balance time provided no discriminatory ability (AUC range:0.42–0.47), poor sensitivity (0.38–0.61) and poor specificity (0.23–0.56).

**Discussion:**

Despite previous observational evidence showing associations between better one-legged balance performance and reduced fall risk, the one-legged balance test had limited prognostic accuracy in predicting recurrent falls. This contradicts ongoing translation of this test into clinical screening tools for falls and highlights the need to consider new and existing screening tools that can reliably predict fall risk.

## Introduction

The two most common recommendations in clinical fall prevention guidelines include exercise interventions and risk stratification screening, specifically assessing fall history in the last year and balance or gait impairments (1, 2). While there is robust evidence demonstrating that functional balance and strength training programmes can reduce fall risk (3, 4), there is limited evidence indicating that a balance or gait assessment, in isolation or in combination with fall history, can accurately predict fall risk (2, 5–7). One commonly used screening tool is the one-legged balance test, commended for its parsimony and low-cost (8–11). The adoption of the test in clinical and population settings is primarily based on evidence from observational studies demonstrating that better balance performance is associated with decreased risk of falls (7, 12–14).

However, a recent systematic review highlighted that the evidence examining associations between one-legged balance and fall risk is poor, relying largely on cross-sectional data (7). It concluded that there was insufficient evidence investigating the prognostic ability of the one-legged balance test in accurately predicting falls. This is concerning for ongoing translation, given that a population-level association does not necessarily equate with prediction at an individual level (15–17). Although successful balance strategies that involve proactive or reactive adaptations are directly involved in avoiding a fall (18–20), there is insufficient investigation as to whether poor one-legged balance performance predicts subsequent falls and what constitutes the threshold for a positive screening result (7).

History of falls is considered the most accurate indicator of future fall risk (1). It is unclear if a balance assessment provides additional information on fall risk, beyond what is already indicated by a simple question on previous falls. Therefore, the aims of this study, using repeat data from a birth cohort study, were to (i) assess the prognostic ability of the one-legged balance test to predict falls; (ii) compare the prognostic accuracy of the one-legged balance test with self-reported fall history; and (iii) identify and assess the optimal cut-points of one-legged balance test times in predicting falls. We also examined differences between eyes open and eyes closed tests, single and recurrent fall outcomes and by age.

## Materials and methods

### Study sample and ascertainment of balance and falls

The Medical Research Council National Survey of Health and Development is an ongoing birth cohort study of 5,362 individuals born within one week in March 1946 (21, 22). At ages 53 and 60–64 years, individuals were asked to complete tests of their balance ability as part of a physical capability assessment. This involved asking them to cross their arms across their chest, stand on one leg and raise the other leg off the ground. Individuals completed one *eyes open* and one *eyes closed* trial. Research nurses stopped timing when the participant's suspended foot touched the ground or 30 s had elapsed. Reasons were documented for those unable to participate in the test (e.g., timer failure, health reasons, etc.). At ages 53, 60–64 and 68, individuals were asked if they had fallen within the last 12 months. Individuals who responded affirmatively were subsequently asked how many times they had fallen; this was recorded on a continuous scale at ages 60–64 and 68 and categorically at age 53 (0,1–2, 3+). Given stronger associations between one-legged balance performance and recurrent rather than single falls in this cohort (23), we focused on recurrent falls (0–1 vs. 2+ falls) as the main outcome. Results for any fall (0 vs. 1+ falls) are presented in Supplementary material. Up to 2,508 participants with data on at least one measure of balance time and reported falls at a follow-up wave were included; detailed information on sample size including those who completed balance assessments and fall questionnaires at each age is documented elsewhere (23).

### Statistical analysis

Analyses were conducted in two stages: (1) calculation of areas under the receiver operating characteristics (ROC) curve and (2) identification of optimal balance time cut-points. For each part, we examined *eyes open* and *eyes closed* balance times across four different combinations of time points: balance age 53 → recurrent falls (0–1 vs. 2+ falls) age 60–64; balance age 53 → recurrent falls age 68; balance age 60–64 → recurrent falls age 68; and balance ages 53 and 60–64 → recurrent falls age 68. Note that the fourth combination included two repeated assessments of balance (at ages 53 and 60–64) to assess if change in balance performance over time was informative for fall risk. Given no previous evidence of sex differences in association between one-legged balance performance and falls in this study population (23), males and females were included in the same model, with sex included as a covariate.

In the first stage, areas under the receiver operating characteristics (ROC) curve (AUC) (24) were calculated to assess the ability of both balance trials (i.e., *eyes open, eyes closed*) to predict falls. An AUC of 0.5 indicates no discriminatory ability, <0.6 poor, 0.6–0.7 average, 0.7–0.8 good, 0.8–0.9 very good, and greater than 0.9 excellent (25). Across each combination of time points and visual condition, we examined four different model progressions (see Supplementary Table S1 for all 32 models). The AUC of the initial sex-only model (Model 1) was compared to a model with sex and balance time (Model 2) and then to a model with sex and fall history (Model 3). Next, the combined model of sex, balance and fall history (Model 4) was compared to the model with sex and balance (Model 2) and the model with sex and fall history (Model 3).”

In the second stage of analyses, optimal cut-points for each balance measure and time point were estimated using the Liu method, which identifies the cut-point that maximises the product of the sensitivity (proportion of individuals with an affirmative fall outcome that had a positive screening result- e.g., above the cut-point) and specificity (proportion of individuals who did not fall who had a negative screening result- e.g., below the cut-point) (26). The cut-point and confidence intervals were obtained using a bootstrap approach with 1,000 iterations, and sensitivity, specificity and AUC for each cut-point are also provided.

Several sensitivity analyses were conducted. First, all analyses were repeated using any fall (0 vs. 1+) as an outcome. Next, we calculated AUC for a binary measure indicating inability to participate in balance test due to health reasons. Finally, optimal cut-points were also estimated using the Youden (cut-point that maximises the sum of the sensitivity and specificity) and the “Closest to (0,1)” (cut-point that minimises the distance to the upper left corner of the AUC - e.g., the point of perfect prediction) methods (27).

## Results

Individuals balanced on one leg with their *eyes open* for a median of 30 s (quartile 1: 28 s, quartile 3: 30 s; 74.0% achieved 30 s maximum) and 30 s (12.5 s, 30 s; 52.5%) at ages 53 and 60–64, respectively, and with their *eyes closed* for 5 s (3, 9; 3.5%) and 3 s (2, 5; 1.0%). At age 53, 1.6% (*n* = 37) of individuals were unable to complete the balance tests due to health reasons, rising to 3.9% (*n* = 85) at age 60–64. Prevalence of recurrent falls was 7.7% (*n* = 169) at age 60–64 and 10.0% (*n* = 225) at age 68; prevalence of any fall was 18.1% (*n* = 396) and 21.7% (*n* = 487), respectively. At age 53, 13.5% reported falling 1 or 2 times and 3.1% reported falling 3+ times.

### Prognostic accuracy of continuous balance measures

**Figure 1:**
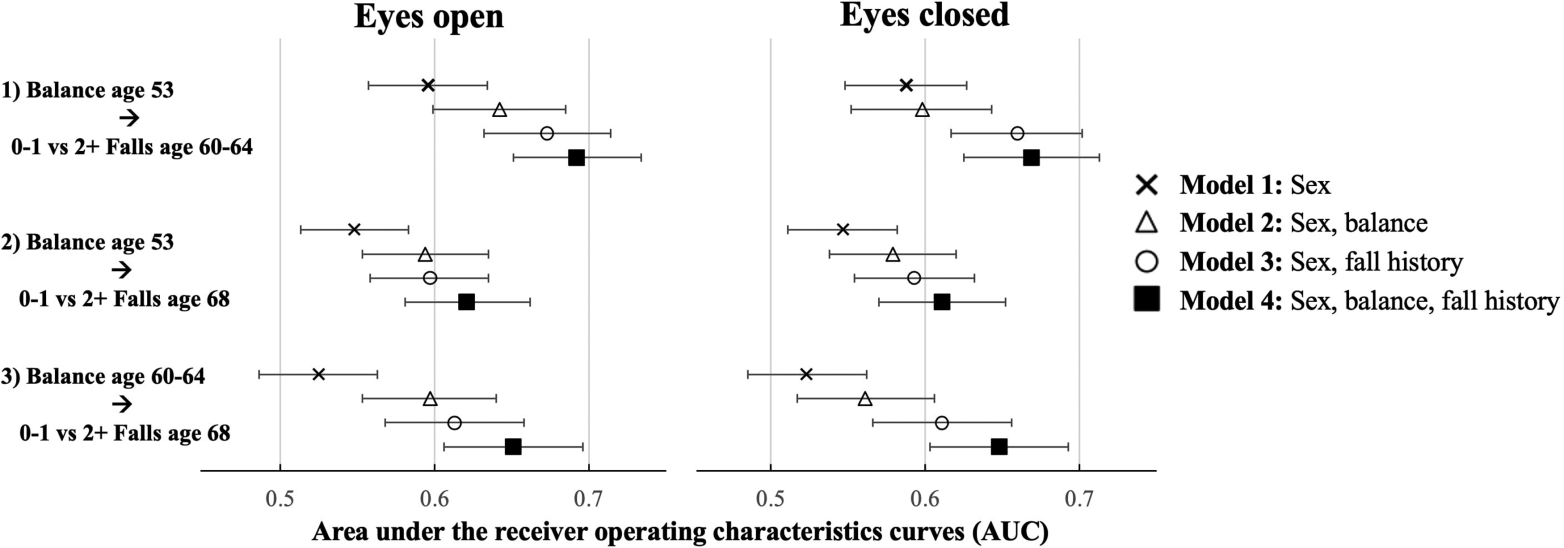
Prognostic accuracy of the one-legged balance test and recurrent falls: comparison of a sex-only model (1); with a balance and sex-adjusted model (2), a sex and past falls model (3) and a sex, balance and past falls model (4) using area under receiver operating characteristics curves (AUC) with eyes open and eyes closed. Note each plotted point refers to a distinct model (32 total).

Figure 1 provides a visual representation of all AUC discussed below; Supplementary Table S1 provides estimates and corresponding p-values for comparisons. The prognostic accuracy of the sex-only models was poor (Figure 1, Model 1; AUC < 0.600). Adding either *eyes open* or *eyes closed* balance time improved the prognostic accuracy of most models (Model 2), with larger improvements for the *eyes open* balance test than the *eyes closed*. However, AUC remained in the poor to average range (0.561–0.642) across all models. The addition of fall history to the sex-only model (Model 3) had a larger impact on prognostic accuracy than adding balance time as AUC were now largely considered “average” (range: 0.593–0.673). A combined sex, fall history and balance time model had the highest prognostic accuracy (Model 4; range: 0.611–0.692). Notably, tests of AUC comparisons revealed that adding fall history to the sex and balance model improved accuracy of the model at all time points (Model 4 vs. 2). Conversely, adding balance time to the sex and fall history model did not improve accuracy (Model 4 vs. 3), with a single statistically significant improvement seen with the addition of balance time with *eyes open* at age 53 in relation to fall risk at age 60–64 (see Supplemental Table 1). The highest AUC was obtained in this model as well (Figure 1; Model 4; 0.692 (0.651, 0.734)).

### Identifying optimal cut-points

Using the Liu method, optimal cut-points ranged from 27 s (25.7, 28.3) to 29 s (20.9, 30) for *eyes open* balance times and from 3 s (2.1, 3.9) to 5 s (3.9, 6.1) for *eyes closed* times (see Table 1). However, across both visual conditions and all time points, AUC were <0.50, suggesting the cut-points provided no discriminatory ability. Sensitivity ranged from 0.38 to 0.61 and specificity ranged from 0.23 to 0.56. The optimal cut-point for number of previous falls was 0 (i.e., positive screening result indicated by any fall); specificity was high (>0.85), but sensitivity (≤0.41) and overall prognostic accuracy (AUC ≤ 0.63) remained low.

**Table 1:** Identifying optimal cut-points for the one-legged balance test (and number of previous falls) in predicting recurrent (0–1 vs. 2+) falls using the Liu method.

	Optimal cut- point (sec; 95% CI)^a^	AUC	Sensitivity	Specificity
1. Balance with eyes open				
Age 53 → Falls age 60–64	28 (26.4, 29.6)	0.42	0.61	0.24
Age 53 → Falls age 68	27 (25.7,28.3)	0.42	0.61	0.23
Age 60–64 → Falls age 68	29 (20.9, 30)	0.42	0.38	0.46
2. Balance with eyes closed				
Age 53 → Falls age 60–64	4 (1.9, 6.1)	0.47	0.49	0.46
Age 53 → Falls age 68	5 (3.9, 6.1)	0.47	0.38	0.56
Age 60–64 → Falls age 68	3 (2.1, 3.9)	0.46	0.44	0.48
3. Number of previous falls^b^				
Age 53 → Falls age 60–64	0	0.62	0.39	0.86
Age 53 → Falls age 68	0	0.57	0.29	0.85
Age 60–64 → Falls age 68	0	0.63	0.41	0.85

AUC, area under receiver operating characteristic curve.

^a^ Lower limit of 95% CI was capped at 0, upper limit was capped at 30 s due to the minimum and maximum scores of the test; however some estimations were below or above these times.

^b^ Fall history at age 53 was only available as a categorical variable (0,1–2,3–11,12+), but considered continuously at age 60–64

### Sensitivity analyses

Prognostic accuracy was lower across all models when considering any fall (0 vs. 1+) compared with recurrent falls (2+) (see Supplementary Table S2). Similarly, there was no evidence to suggest that inability to complete a balance test due to health reasons accurately predicted falls, with AUC consistently lower than the *eyes open* and *eyes closed* tests across all models (see Supplementary Table S3). The “Closest to (0,1)” method identified identical cut-points to those identified by the Liu method (see Supplementary Table S4). However, the Youden method identified balance cut-points that maximised sensitivity or specificity (e.g., 1) at the expense of minimal corresponding sensitivity or specificity (e.g., 0); for example, “optimal” cut-points were identified as 0 s (i.e., any time on the balance test) or 30 s (i.e., completed balance test). Cut-points across the three methods remained similar when any fall was considered as the outcome (see Supplementary Table S5).

## Discussion

Using data from a representative observational cohort study with balance and falls assessments between ages 53 and 69 years, we found poor prognostic accuracy of one-legged balance tests in predicting falls over four to fifteen years of follow-up. Self-reported fall history was a better predictor of future falls compared with one-legged balance performance, however, discriminatory ability remained below average. The identification and application of “optimal” binary balance cut-points showed no ability to discriminate recurrent fallers or any fallers from non-fallers. Results were consistent across *eyes open* and *eyes closed* conditions. Findings emphasise the immediate need for caution against ongoing translation of the one-legged balance test into clinical screening tools for fall risk, particularly with absolute cut-off times, and highlight the necessity for more research in this area.

The findings presented in this study are consistent with other longitudinal studies examining prediction of any fall (AUC ≤ 0.56) (7, 28–31), although no study examined recurrent fall outcomes. Of note, these studies reported higher specificity (range: 46.2–90.3%) compared with sensitivity (16.7–83.5%), a pattern not observed in our study. Although continuous one-legged balance time demonstrated some, albeit poor to average, prognostic accuracy, the application of binary cut-points negated any indication of reliable predictive ability. The “optimal” cut-points of both the *eyes open* and *eyes closed* tests were similar to the median balance scores, resulting in nearly half the sample having a positive screening result. Therefore, it was unsurprising that such cut-points lacked discriminatory ability, with poor sensitivity and specificity. The systematic review (7) identified fifteen different cut-points (ranging from 1 s (32) to 55 s (33)), most commonly using 5 s, however, there was no empirical evidence to support any of these. It is plausible that repeated indicators of balance performance over time could provide clinical value if continuous performance is considered in relation to age and sex during a clinical assessment, but this must be formally assessed in clinical settings. Nevertheless, results strongly indicate that current translation of binary cut-points into screening provides no useful information. Prevalence of any falls reported in this study was comparable with pooled prevalence estimates from harmonised data from NSHD and three other studies aged 50–54 (men: 13.4%, women: 20.9%) and 60–64 years (men: 15.7%, women: 29.9%) (34); unfortunately recurrent fall prevalence was not reported and so comparison for this outcome is not possible.

Replacing or combining one-legged balance performance with a self-reported measure of fall history improved prognostic fit from poor to average. Specificity of previous fall history was high (≥0.85), indicating that fall history may be a reliable screening option to identify those requiring interventions to avoid subsequent falls. However, reliance on fall history dictates that screening would only be beneficial after a fall has occurred, and therefore, it has limited utility as a screening tool aiming to prevent falls. There was some evidence to suggest that larger improvements in the prognostic accuracy were observed when adding eyes open balance time compared with eyes closed time. Given that the majority of the sample achieved the full 30 seconds with eyes open, an inability to complete the eyes open test at such a young age may highlight an increased risk of falls that is not as evident in the eyes closed test where lower scores are more common. This is supported by high cut-points identified for the eyes open tests (range: 27–29 s) and lower cut-points for the eyes closed test (range: 3–5 s).

The average prognostic performance of combining sex, one-legged balance assessment and self-reported fall history further highlights the complexity of fall risk. A single parsimonious assessment such as the one-legged balance test, including repeat measurements over a 10-year period, may not be a sufficient screening tool in community-dwelling samples (5, 7, 35). One study has suggested that fall screening tools with fewer than five predictors are suboptimal, recommending that 20–30 items are ideal to maximise predictive accuracy (36). While parsimony is often the aim, a multifactorial assessment tool for balance – similar to the Framingham Risk Score for cardiovascular risk (37)– may provide the most accurate risk prediction. Further research is necessary to identify a tool that provides the optimal combination of parsimony and accuracy.

Although this study highlights the poor effectiveness of one-legged balance performance as a screening tool, exploring one-legged balance may still be helpful for understanding mechanistic associations between balance and falls. For example, the ability to adequately maintain one's balance is linked with successful engagement in strategic mechanisms that prevent falling such as anticipatory ankle and hip adjustments for small perturbations, stepping strategies for larger disturbances, and postural rescue strategies (e.g., grabbing an object, extending arm etc.) (18, 19). Additionally, evidence consistently shows that interventions targeting balance exercises effectively reduce fall risk confirming a clear role of balance ability in fall mechanisms (3, 4). Previous investigation in this cohort has identified relatively strong and consistent population level associations between poorer one-legged balance performance and increased odds of recurrent falls, which were sustained after adjustment for socioeconomic, health, behavioural or cognitive factors (23). However, these new findings demonstrate that this does not translate to prediction of falls at an individual level. This serves to highlight the need for caution when developing clinical screening guidelines as it is a clear example of the fact that a strong statistical association at the population level does not equate with good individual level prediction (15–17).

Key strengths of this study include the large, population-representative study sample, repeat ascertainment of balance performance, and the comprehensive analysis across multiple one-legged balance measures (*eyes open*, *eyes closed*, *inability to do test due to health reasons*), fall outcomes (recurrent, any) and time points (ages 53, 60–64 and 68; combined measures at ages 53 and 60–64). There are some limitations. Due to financial and logistical constraints of any large cohort study, fall outcomes were self-reported, and fall severity could not be ascertained. AUC may also be lower than expected due to death as a competing risk. Given that clinical fall risk screening may focus on shorter prevention windows, the long intervals between balance assessment and fall outcomes (i.e., 4–15 years) may have weakened associations and, therefore must be interpreted with caution. For example, balance performance at age 53 years was the strongest predictor of mortality when compared with other physical capability measures in NSHD (38) and therefore individuals with poor balance ability, who would have been at the highest risk of falls, would have been more likely to die before follow-up of falls data at age 60–64 or 68. Similar to blood pressure variability (39), it is possible that balance performance on the test day was not reflective of an individual's true performance. For example, any single balance performance may be impacted by acute health conditions, psychological factors, or other extenuating circumstances. Finally, one-legged balance was assessed by a trained research nurse with a timer; a more sensitive measure of balance, including single and double-legged balance on a force plate, may have greater success in identifying those at greatest risk of falls (13, 40).

Despite previous evidence of an observational association between one-legged balance and recurrent falls in this cohort (23), this study highlighted that one-legged balance performance was a poor prognostic indicator of subsequent fall risks over a four to fifteen year period. Further research is needed to examine how empirical associations can be translated into effective screening tools to address problems encountered by the ageing population. Unfortunately, poor translation and communication of one-legged balance research (11) is common and has resulted in premature translation of the test into clinical settings to predict fall risk. There is an urgent need for replication of study findings to encourage caution in translation of evidence, and a need for further research to assess the prognostic ability of other viable fall risk screening tools.

## Data Availability

The datasets presented in this article are not readily available because access to NSHD data adheres to strict confidentiality guidelines. These data are available to bona fide researchers upon request to the NSHD Data Sharing Committee via a standard application procedure. doi: https://doi.org/10.5522/NSHD/Q101; doi: https://doi.org/10.5522/NSHD/Q102; doi: https://doi.org/10.5522/NSHD/Q103. Requests to access the datasets should be directed to http://www.nshd.mrc.ac.uk/data.
