# Supplementary material for: Prognostic accuracy of the one-legged balance test in predicting falls: 15-years of midlife follow-up in a British birth cohort study
Source: Front Sports Act Living. 2023 Jan 9;4:1066913. doi: 10.3389/fspor.2022.1066913 (PMC9869374; doi:10.3389/fspor.2022.1066913)
Supplement: Supplementary file 1 [file Datasheet1.pdf]

Supplementary Table 1. Prognostic accuracy of the one-legged balance test and **recurrent (2+)** falls: comparison of a sex only model (A); a balance and sex-adjusted model (B); a sex and past falls model (C) and a sex, balance and past falls model (D) using area under receiver operating characteristics curves (AUC)

**Independent variable:** Temporal association	**Sample size**	**Model A: sex only[a]**	**Model B: sex and balance**	**Test of comp[bc]**	**Model C: sex and fall history**	**Test of comp[bd]**	**Model D: balance, sex and fall history**	**Test of comp[be]**	**Test of comp[bf]**
**1. Balance with eyes open**									
Age 53 → Falls age 60-64	2061	0.596 (0.557, 0.634)	0.642 (0.599, 0.685)	<0.001	0.673 (0.632, 0.714)	<0.001	0.692 (0.651 0.734)	<0.005	0.04
Age 53 → Falls age 68	2123	0.548 (0.513, 0.583)	0.594 (0.553, 0.635)	<0.005	0.597 (0.558, 0.635)	<0.001	0.621 (0.581 0.662)	0.04	0.05
Age 60-64 → Falls age 68	1852	0.525 (0.486, 0.563)	0.597 (0.553, 0.640)	<0.005	0.613 (0.568, 0.658)	<0.001	0.651 (0.606 0.696)	0.01	0.09
Age 53 & 60-64 → Falls age 68	1748	0.536 (0.496, 0.576)	0.610 (0.565, 0.656)	<0.005	0.634 (0.587, 0.681)	<0.001	0.660 (0.613, 0.706)	0.01	0.11
**2. Balance with eyes closed**									
Age 53 → Falls age 60-64	2010	0.588 (0.548, 0.627)	0.598 (0.552, 0.643)	0.44	0.66 (0.617, 0.702)	<0.001	0.669 (0.625 0.713)	<0.001	0.27
Age 53 → Falls age 68	2067	0.547 (0.511, 0.582)	0.579 (0.538, 0.620)	0.02	0.593 (0.554, 0.632)	<0.001	0.611 (0.57 0.652)	0.04	0.10
Age 60-64 → Falls age 68	1846	0.523 (0.485, 0.562)	0.561 (0.517, 0.606)	0.07	0.611 (0.566, 0.656)	<0.001	0.648 (0.603 0.693)	<0.001	0.07
Age 53 & 60-64 → Falls age 68	1696	0.533 (0.493, 0.574)	0.567 (0.520, 0.613)	0.10	0.624 (0.576, 0.672)	<0.001	0.637 (0.588, 0.686)	<0.005	0.42

[a] AUC fluctuates due to minor variations in sample size; sample size is identical for the three models in each row

[b] *p<0.05 signifies that the model with the higher AUC is a significantly better prognostic model.*

[c] *tests equality of the AUC of the sex and balance model with the AUC of sex only model within the same sample.*

[d] *tests equality of the AUC of the sex and balance model with the AUC of sex only model within the same sample.*

[e] *tests the equality of AUC of sex, balance and past falls model with the AUC of the sex and balance model within the same sample.*

[f] *tests the equality of AUC of sex, balance and past falls model with the AUC of the sex and fall history model within the same sample.*

Recall: An AUC greater than 0.9 is considered excellent, greater than 0.8 to 0.9 very good, 0.7 to 0.8 good, 0.6 to 0.7 average, <0.6 poor and ~0.5 indicating no discriminatory ability [25].

Supplementary Table 2. Prognostic accuracy of the one-legged balance test and **any (1+)** falls: comparison of a sex only model (A); a balance and sex-adjusted model (B); a sex and past falls model (C) and a sex, balance and past falls model (D) using area under receiver operating characteristics curves (AUC)

**Independent variable:** Temporal association	**Sample size**	**Model A: sex only**[a]	**Model B: sex and balance**	**Test of comp**[bc]	**Model C: sex and fall history**	**Test of comp**[bd]	**Model D: balance, sex and fall history**	**Test of comp**[be]	**Test of comp**[bf]
**1. Balance with eyes open**									
Age 53 → Falls age 60-64	2066	0.580 (0.553, 0.607)	0.600 (0.570, 0.630)	<0.005	0.625 (0.595, 0.654)	<0.0001	0.634 (0.603, 0.665)	<0.005	0.05
Age 53 → Falls age 68	2130	0.565 (0.540, 0.590)	0.580 (0.552, 0.608)	0.02	0.603 (0.576, 0.630)	<0.001	0.607 (0.579, 0.636)	<0.005	0.33
Age 60-64 → Falls age 68	1855	0.555 (0.528, 0.582)	0.577 (0.546, 0.608)	<0.01	0.608 (0.578, 0.638)	<0.001	0.616 (0.585, 0.648)	<0.001	0.22
Age 53 & 60-64 → Falls age 68	1751	0.560 (0.532, 0.588)	0.580 (0.548, 0.612)	0.01	0.630 (0.599, 0.662)	<0.001	0.632 (0.599, 0.665)	<0.001	0.78
**2. Balance with eyes closed**									
Age 53 → Falls age 60-64	2015	0.579 (0.552, 0.606)	0.587 (0.556, 0.618)	0.34	0.619 (0.589, 0.649)	<0.0001	0.627 (0.595, 0.658)	<0.001	0.23
Age 53 → Falls age 68	2074	0.566 (0.541, 0.592)	0.573 (0.544, 0.603)	0.37	0.604 (0.577, 0.631)	<0.0001	0.604 (0.574, 0.634)	<0.005	0.98
Age 60-64 → Falls age 68	1849	0.555 (0.528, 0.582)	0.577 (0.546, 0.608)	0.01	0.608 (0.577, 0.638)	<0.001	0.620 (0.589, 0.652)	<0.001	0.05
Age 53 & 60-64 → Falls age 68	1699	0.559 (0.531, 0.587)	0.582 (0.550, 0.614)	0.01	0.628 (0.596, 0.659)	<0.001	0.637 (0.605, 0.669)	<0.001	0.08

[a] AUC fluctuates due to minor variations in sample size; sample size is identical for the three models in each row. Sample size differs from Supplementary Table 1 as some individuals who fell in the last year had missing data on the number of falls.

[b] *p<0.05 signifies that the model with the higher AUC is a significantly better prognostic model.*

[c] *tests equality of the AUC of the sex and balance model with the AUC of sex only model within the same sample.*

[d] *tests equality of the AUC of the sex and balance model with the AUC of sex only model within the same sample.*

[e] *tests the equality of AUC of sex, balance and past falls model with the AUC of the sex and balance model within the same sample.*

[f] *tests the equality of AUC of sex, balance and past falls model with the AUC of the sex and fall history model within the same sample.*

Recall: An AUC greater than 0.9 is considered excellent, greater than 0.8 to 0.9 very good, 0.7 to 0.8 good, 0.6 to 0.7 average, <0.6 poor and ~0.5 indicating no discriminatory ability [25].

Supplementary Table 3. Prognostic accuracy of **<u>inability to complete balance test (yes/no indicator)</u>** and risk of recurrent (2+) or any (1+) falls: comparison of a balance and sex-adjusted model to A) a sex only model; B) a sex and past falls model and C) a sex, balance and past falls model using area under receiver operating characteristics curves (AUC)

**Independent variable:** Temporal association	**Sample size**	**Model A: sex only[a]**	**Model B: sex and balance**	**Test of comp[bc]**	**Model C: sex and fall history**	**Test of comp[bd]**	**Model D: balance, sex and fall history**	**Test of comp[be]**	**Test of comp[bf]**
**1. Inability to complete balance test → Recurrent (2+) falls**									
Age 53 → Falls age 60-64	2024	0.591 (0.553, 0.629)	0.611 (0.572, 0.652)	0.03	0.679 (0.638, 0.720)	<0.001	0.680 (0.639, 0.722)	<0.001	0.65
Age 53 → Falls age 68	2080	0.544 (0.508, 0.579)	0.553 (0.517, 0.588)	0.18	0.600 (0.561, 0.638)	<0.001	0.599 (0.560, 0.639)	<0.005	0.39
Age 60-64 → Falls age 68	1904	0.523 (0.486, 0.560)	0.553 (0.513, 0.593)	<0.01	0.629 (0.586, 0.672)	<0.001	0.635 (0.591, 0.678)	<0.001	0.19
Age 53 & 60-64 → Falls age 68	1749	0.532 (0.493, 0.570)	0.565 (0.523, 0.606)	<0.01	0.648 (0.603, 0.693)	<0.001	0.649 (0.603, 0.695)	<0.001	0.76
**. Inability to complete balance test → Any (1+) fall**									
Age 53 → Falls age 60-64	2029	0.580 (0.553, 0.607)	0.586 (0.558, 0.613)	0.23	0.627 (0.597, 0.657)	<0.0001	0.626 (0.596, 0.656)	<0.001	0.49
Age 53 → Falls age 68	2087	0.564 (0.539, 0.589)	0.565 (0.539, 0.590)	0.54	0.607 (0.579, 0.634)	<0.0001	0.606 (0.578, 0.633)	<0.001	0.21
Age 60-64 → Falls age 68	1909	0.552 (0.525, 0.578)	0.571 (0.544, 0.598)	<0.005	0.613 (0.583, 0.642)	<0.001	0.620 (0.590, 0.649)	<0.001	0.08
Age 53 & 60-64 → Falls age 68	1754	0.556 (0.528, 0.583)	0.577 (0.548, 0.605)	<0.005	0.638 (0.607, 0.669)	<0.001	0.640 (0.609, 0.671)	<0.001	0.47

[a] AUC fluctuates due to minor variations in sample size; sample size is identical for the three models in each row. Sample size differs from Supplementary Table 1 as some individuals who fell in the last year had missing data on the number of falls.

[b] *$p<0.05$ signifies that the model with the higher AUC is a significantly better prognostic model.*

[c] *tests equality of the AUC of the sex and balance model with the AUC of sex only model within the same sample.*

[d] *tests equality of the AUC of the sex and balance model with the AUC of sex only model within the same sample.*

[e] *tests the equality of AUC of sex, balance and past falls model with the AUC of the <u>sex and balance model</u> within the same sample.*

[f] *tests the equality of AUC of sex, balance and past falls model with the AUC of the <u>sex and fall history model</u> within the same sample.*

Recall: An AUC greater than 0.9 is considered excellent, greater than 0.8 to 0.9 very good, 0.7 to 0.8 good, 0.6 to 0.7 average, <0.6 poor and ~0.5 indicating no discriminatory ability [25].

Supplementary Table 4. Identifying optimal cut-points of the one-legged balance test (and number of previous falls) in predicting recurrent (0-1 vs 2+) falls using the Closet to (0,1) and Youden methods

	Optimal cut- point (sec; 95% CI)[a]	*AUC*	*Sensitivity*	*Specificity*
**CLOSEST TO (0,1) METHOD**				
**1. Balance with eyes open**				
Age 53 → Falls age 60-64	28 (23.6, 30)	0.42	0.61	0.24
Age 53 → Falls age 68	27 (24.9, 29.1)	0.42	0.61	0.23
Age 60-64 → Falls age 68	29 (18.0, 30)	0.42	0.37	0.46
**2. Balance with eyes closed**				
Age 53 → Falls age 60-64	4 (1.7, 6.3)	0.47	0.49	0.46
Age 53 → Falls age 68	5 (3.7,6.3)	0.47	0.38	0.56
Age 60-64 → Falls age 68	3 (2.1, 3.9)	0.45	0.40	0.49
**3. Number of previous falls**[b]				
Age 53 → Falls age 60-64	0 (n/a)	0.62	0.39	0.86

Age 53 → Falls age 68	0 (n/a)	0.57	0.29	0.85
Age 60-64 → Falls age 68	0 (n/a)	0.63	0.41	0.85
**YOUDEN METHOD**				
**1. Balance with eyes open**				
Age 53 → Falls age 60-64	1 (0, 3.6)	0.5	1	0.01
Age 53 → Falls age 68	0 (0, 21.6)	0.50	1	0
Age 60-64 → Falls age 68	30	0.50	0	1
**2. Balance with eyes closed**				
Age 53 → Falls age 60-64	0 (0, 8.5)	0.5	1	0
Age 53 → Falls age 68	0 (0, 8.6)	0.5	1	0
Age 60-64 → Falls age 68	30 (5.0, 30)	0.5	0	1
**3. Number of previous falls**[b]				
Age 53 → Falls age 60-64	0 (n/a)	0.62	0.39	0.86
Age 53 → Falls age 68	0 (n/a)	0.57	0.29	0.85
Age 60-64 → Falls age 68	0 (n/a)	0.63	0.41	0.85

AUC= area under receiver operating characteristic curve

[a]Lower limit of 95% CI was capped at 0, upper limit was capped at 30s due to the minimum and maximum scores of the test; however some estimations were below or above these times.

[b]Fall history at age 53 was only available as a categorical variable (0,1-2,3-11,12+), but considered continuously at age 60-64

Supplementary Table 5. Identifying optimal cut-points of the one-legged balance test (and number of previous falls) in predicting **any (1+)** falls using the Liu, Youden and Closet to (0,1) methods

	Optimal cut- point (sec; 95% CI)[a]	*AUC*	*Sensitivity*	*Specificity*
**LIU METHOD**				
**1. Balance with eyes open**				
Age 53 → Falls age 60-64	28 (26.6, 29.4)	0.46	0.68	0.23
Age 53 → Falls age 68	29 (27.8, 30)	0.47	0.69	0.25
Age 60-64 → Falls age 68	29 (24.2, 30)	0.45	0.45	0.46
**2. Balance with eyes closed**				
Age 53 → Falls age 60-64	4 (2.5, 5.5)	0.47	0.49	0.45
Age 53 → Falls age 68	5 (4.5, 5.5)	0.50	0.43	0.57
Age 60-64 → Falls age 68	3 (2.4, 3.6)	0.46	0.43	0.48
**3. Number of previous falls** [b]				
Age 53 → Falls age 60-64	0	0.58	0.29	0.87
Age 53 → Falls age 68	0	0.56	0.26	0.86
Age 60-64 → Falls age 68	0	0.59	0.32	0.86
**CLOSEST TO (0,1) METHOD**				
**1. Balance with eyes open**				
Age 53 → Falls age 60-64	28 (25.8, 30)	0.50	0.68	0.23
Age 53 → Falls age 68	29 (27.5, 30)	0.47	0.69	0.25
Age 60-64 → Falls age 68	29 (23.6, 30)	0.45	0.45	0.46
**2. Balance with eyes closed**				
Age 53 → Falls age 60-64	4 (2.5, 5.5)	0.47	0.49	0.45
Age 53 → Falls age 68	5 (4.7, 5.3)	0.50	0.43	0.57
Age 60-64 → Falls age 68	3 (2.3, 3.7)	0.46	0.43	0.48
**3. Number of previous falls** [1]				
Age 53 → Falls age 60-64	0	0.58	0.29	0.87
Age 53 → Falls age 68	0	0.56	0.26	0.86
Age 60-64 → Falls age 68	0	0.59	0.32	0.86
**YOUDEN METHOD**				
**1. Balance with eyes open**				
Age 53 → Falls age 60-64	1 (0, 6.6)	0.50	1	0.01
Age 53 → Falls age 68	1 (0, 20.00)	0.50	0.99	0.01
Age 60-64 → Falls age 68	30	0.50	0.00	1
**2. Balance with eyes closed**				
Age 53 → Falls age 60-64	1 (0, 8.2)	0.50	0.97	0.04
Age 53 → Falls age 68	0 (0, 11.1)	0.50	1	0.00
Age 60-64 → Falls age 68	30 (6.3, 30)	0.50	0.00	1
**3. Number of previous falls** [b]				
Age 53 → Falls age 60-64	0	0.58	0.29	0.87
Age 53 → Falls age 68	0	0.56	0.26	0.86
Age 60-64 → Falls age 68	0	0.59	0.32	0.86

AUC= area under receiver operating characteristic curve

[a]Lower limit of 95% CI was capped at 0, upper limit was capped at 30s due to the minimum and maximum scores of the test; however some estimations were below or above these times.

[b]Fall history at age 53 was only available as a categorical variable (0,1-2,3-11,12+), but considered continuously at age 60-64
